# Special Issue “Viral Evasion or Suppression of Host Immunity”

**DOI:** 10.3390/v12060656

**Published:** 2020-06-18

**Authors:** Bumsuk Hahm

**Affiliations:** Departments of Surgery and Molecular Microbiology & Immunology, University of Missouri, Columbia, MO 65212, USA; hahmb@health.missouri.edu

Viruses have evolved to survive in hosts, presumably by devising meticulous strategies to elude or suppress host immunity. It is crucial to understand the interplay between viruses and host immunity from scientific and clinical standpoints. 

In this Special Issue, several original research articles demonstrated the intriguing methods which immune evasion viruses utilize for their survival and pathogenicity. Dubrow et al. determined the structural basis by which the NS1 protein of the highly pathogenic 1918 influenza A virus hijacks the cellular proteins of PI3K p85β and CRK (CT-10 regulator of kinase) to promote viral propagation [1]. They demonstrated that 1918 NS1 increases its affinity with PI3K p85β in the presence of CRK, which may lead to enhanced PI3K activation and heightened viral pathogenicity. Cheng et al. explored the mechanism by which non-cytopathic bovine viral diarrhea virus (BVDV) escapes the host’s innate immunity, leading to early pregnancy failure in cows [2]. They found that BVDV inhibited tau-induced interferon stimulated gene expression from uterine endometrial cells. Wang et al. identified UL24 of pseudorabies virus (PRV) as a viral protein that counteracts host immunity by inducing the degradation of p65, an essential component for NF-κB activation [3]. The authors generated UL24-deficient PRV, which was used fittingly as a control for their findings. Gao et al. determined neutralization antibody specificities in macaques which developed neutralization breadth after long-term simian/human immunodeficiency virus infection and identified neutralization escape mutations [4]. Zhu et al. found that following simian 28 type D retrovirus (SRV) infection, the autophagic proteins LC3 and p62/SQSTM1 interacted with procaspase-8, linking cellular autophagy to the apoptosis pathway in Jurkat T cells [5]. This could be a potential pathogenetic mechanism for the loss of T lymphocytes which has been observed during SRV infection. Interestingly, Lim et al. demonstrated that omega-3 (*n*-3) polyunsaturated fatty acids (PUFAs) interfere with MHC-TCR interactions in CD8^+^ T cells [6]. In this study, highly inclined and laminated optical sheet microscopic analysis was used to uncover how TCR motility was reduced on the anti-viral CD8^+^ T cells that endogenously synthesize *n*-3 PUFAs. 

Interesting review articles were reported to emphasize virus–host immunity interplay. These include the hepatitis B virus-mediated suppression of the host’s innate immunity [7], a summary of the viral and host factors contributing to the pathogenesis of hypervirulent fowl adenovirus serotype 4 (FAdV-4) strains that cause hepatitis-hydropericardium syndrome [8] and the interaction between gut microbiota and viral infections and their impact on immune regulations in chicken, where four viral diseases, including Avian influenza, were discussed [9]. Furthermore, our group has summarized recent findings on sphingosine 1-phosphate (S1P)-metabolizing enzymes that regulate host defense and immunity to virus infections [10]. Of particular note, Alhammad and Fehr reported that several viruses, including coronaviruses, utilize the macrodomain as a unique mechanism to counter hosts’ antiviral responses via the regulation of ADP-ribosylation [11].

Collectively, these reports exemplify the importance of virus–host immunity interplay and provide new avenues for exciting virus research. 
